# Anesthesia for Pediatric Deep Brain Stimulation

**DOI:** 10.1155/2010/401419

**Published:** 2010-08-10

**Authors:** Joseph Sebeo, Stacie G. Deiner, Ron L. Alterman, Irene P. Osborn

**Affiliations:** ^1^Department of Anesthesiology, Mount Sinai School of Medicine, One Gustave L. Levy Place, New York, NY 10029, USA; ^2^Department of Neurosurgery, Mount Sinai School of Medicine, One Gustave L. Levy Place, Box 1136, New York, NY 10029, USA

## Abstract

In patients refractory to medical therapy, deep brain stimulations (DBSs) have emerged as the treatment of movement disorders particularly Parkinson's disease. Their use has also been extended in pediatric and adult patients to treat epileptogenic foci. We here performed a retrospective chart review of anesthesia records from 28 pediatric cases of patients who underwent DBS implantation for dystonia using combinations of dexmedetomidine and propofol-based anesthesia. Complications with anesthetic techniques including airway and cardiovascular difficulties were analyzed.

## 1. Introduction

Dystonia is a movement disorder characterized by abnormal repetitive and twisting movements resulting in painful and debilitating postures [1]. Dystonia is termed primary when it is idiopathic or may be secondary to a variety of diseases including stroke, cerebral palsy, and neurodegenerative diseases. The pathophysiology of dystonia is not well understood though secondary dystonias often involve injury to the globus pallidus (GP) [2], and electrophysiologic recordings from GP pars interna (GPi) neurons of dystonic patients show abnormal activity [3]. There is no medical cure for dystonia. Medical therapy with anti-Parkinsonian, antispasmodic, or anticholinergic drugs is targeted at symptomatic relief but is only marginally effective [4]. Surgical treatments for dystonia traditionally involved lesioning thalamic nuclei or the pallidum [5] and were associated with a high incidence of complications, including hemiparesis and dysarthria [6]. With the advent of DBS, surgeons have turned to this new surgical approach. 

Deep brain stimulation (DBS) has emerged as the treatment of choice for adult movement disorders that are refractory to medical therapy. This includes DBS of the subthalamic nucleus (STN) or GPi for Parkinson's disease (PD) [7, 8] and DBS of the ventrolateral thalamus for the treatment of essential tremor [9]. As opposed to pallidotomy, DBS is less destructive of brain tissue, is associated with fewer cognitive and motor side effects, and is reversible and adjustable. Based on the evidence involving the GPi in the pathophysiology of dystonia and based on clinical observations that pallidal DBS alleviates off-medication dystonia in PD patients, the GPi has become the target of DBS for dystonia patients with significant clinical improvements [10–12].

The anesthetic challenges during DBS include providing sedation and analgesia with minimal respiratory depression or interference with electrophysiologic brain mapping and clinical testing. Intraoperative cardiovascular hemodynamics and blood pressure control are also important, as a potential complication of DBS procedures is intracerebral hemorrhage, which may occur in 2%–4% of cases [13]. The *α*-2 agonist dexmedetomidine (Dex) is a unique anesthetic agent which can provide sedation, analgesia, and decreased arterial blood pressure without respiratory depression over a broad range of doses, making it a potentially ideal sedative for DBS surgery [14]. These properties have led to the current introduction of Dex-based anesthesia as an alternative to the propofol/fentanyl anesthetic technique in PD patients who require intraoperative sedation [15]. 

Dex has also recently been reported for use in pediatric patients during neurosurgery [16, 17]. Here we present a retrospective study of the use of Dex and propofol anesthesia during DBS surgery in 28 pediatric cases from dystonia patients ranging from 7 to 17 years of age. 

## 2. Materials and Methods

After obtaining IRB approval, the anesthetic records from 28 pediatric DBS cases were reviewed. The following technique was used.

In the preoperative holding area, patients received local anesthesia (lidocaine plus bupivacaine) for placement of the stereotactic head frame (Leksell Model G, Elekta Inc, Atlanta, GA), supplemented with fentanyl and propofol boluses as needed. Patients received propofol infusions for the subsequent localizing MRI. Once the MRI was completed, patients were brought to the OR, where standard anesthesia monitoring (noninvasive arterial blood pressure cuff, pulse oximetry, electrocardiogram, and bispectral index monitor (BIS)) was applied. 

Sedation was continued with propofol and maintained with propofol infusions plus small boluses of fentanyl as needed until scalp incision. Local anesthesia, consisting of 0.5% bupivacaine, was injected into the supraorbital and greater occipital nerves to produce a “scalp block”. Sedation was maintained during scalp incision and burr-hole placement and until the time of intraoperative testing, using a combination of Dex infusion (0.2 to 0.9 *μ*g/kg/hr) and propofol boluses and/or infusions (rates of 15–100 *μ*g/kg/min). BIS monitoring was used with target values >70. Following intraoperative testing, small doses of fentanyl and/or midazolam were administered as needed for discomfort. Following surgery, each patient underwent a second MRI to check electrode placement with anesthesia provided by propofol infusion. 

Our pediatric patients ranged from 7 to 17 years of age, and included both males and females suffering from primary or secondary dystonia. Demographic data and clinical characteristics are summarized in Table 1. Anesthetic records from these patients were analyzed for medications used during surgery and potential intraoperative complications. 

## 3. Results

Our analysis is comprised of 28 consecutive cases of pallidal DBS surgery for dystonia in pediatric patients. Table 2 summarizes the medications given intraoperatively including both the average dose and range. The table displays the data for the entire procedure (MRI plus procedure) and also for the implant procedure alone. Indeed, this table describes the relative use and dose of each anesthetic agent (as bolus or infusion) during intraoperative DBS and compares it to the values used during the entire case. Propofol was used in 27 cases for the entire procedure with an average total dose of 258.6 mg, while it was used in 24 of the cases during the DBS part of the procedure with an average total dose of 115.2 mg. A scalp block was performed in 7 cases. 

Table 3 displays the surgical complications in our cases. No airway difficulty, respiratory depression, hypoxemia, or unplanned conversion to general anesthesia was encountered in any of the cases. In addition, no seizures occurred, and patients remained calm during the entirety of the surgery. One patient suffered a venous air embolus following burr-hole placement. The patient started to cough and complained of chest pain. The head was lowered and bone wax applied. The patient improved but the procedure was aborted. One month later, he underwent successful DBS surgery. Our rate of venous embolism (1 out of 28 cases or 3.6%) is very similar to the rate reported in the literature for DBS surgeries [18]. Venous emboli are a known potential complication of DBS, and although rare, they should always be considered when the patient begins to cough suddenly during the operative phase [18].

## 4. Discussion

We here describe a retrospective study examining anesthetics used for DBS surgery in pediatric dystonia patients. As previously mentioned, awake neurosurgery presents unique anesthetic challenges. Different techniques have been developed to meet this challenge, with propofol infusion alone or in combination with fentanyl/remifentanil the method most frequently reported [19, 20]. However, the use of these anesthetic techniques may be accompanied by complications such as airway obstruction, deep sedation, and respiratory depression [21, 22]. 

Dex is a central *α*-2 adrenergic stimulant and is known to have dose-reducing effects when used with inhalational anesthetics and opiates [23]. Dex offers some unique anesthetic characteristics as it provides sedation and analgesia while minimizing respiratory depression even at large doses [24]. Therefore, Dex is ideally suited for the “asleep-awake-asleep” technique often required for awake craniotomy [25]. With this technique, patients are anesthetized or sedated for the initial craniotomy then allowed to wake up to participate in neurocognitive mapping, and the original anesthesia or sedation is resumed for the final part of the procedure [17, 25]. Sedation with Dex leaves a patient sleepy but still arousable to an alert state, which is very useful for intraoperative recording [25]. 

The anesthetic challenges during DBS surgery are to alleviate the stress of awake neurosurgery without interfering with neurophysiological monitoring or inducing respiratory depression. We therefore employed a similar “asleep-awake-asleep” anesthesia technique for pediatric dystonia patients using a combination of propofol and Dex-based anesthesia. In this way, anesthesia was performed with a high degree of safety and minimal side effects or intraoperative complications. All patients tolerated the procedure well and appeared comfortable. BIS monitoring helped in guiding the anesthetic need with a target of 70–90 for the “awake” part of the surgery. 

Seven of the patients received a “scalp block”, which provides regional anesthesia to the nerves of the scalp and may include the supraorbital, supratrochlear, greater, and lesser occipital nerves, the auriculotemporal nerves, and the greater auricular nerves. Infiltration of these nerves with the local anesthetic bupivacaine blunts hemodynamic responses during craniotomy in both adult [26] and pediatric patients [27]. Although quantification of postoperative pain was not available in the studied anesthetic records, scalp blocks using the local anesthetic ropivacaine have previously been shown to decrease pain severity following craniotomy in adults [28]. 

Dex and propofol have been used successfully in DBS for adult patients suffering from PD [15] and in both pediatric and adult patients undergoing awake craniotomy for epileptogenic focus resection [29]. Similarly, we here report that the use of propofol in conjunction with Dex during DBS for pediatric dystonia patients is safe, efficacious, and well tolerated. The advantages of propofol include both rapid onset of anesthesia and rapid awakening. It is also important to allow patients to interact during DBS as provided by Dex, with sedation coupled with minimal respiratory depression. This enables clinicians to bond with patients, reducing anxiety and fear, to assess efficacy of DBS testing, and to detect any side effects from stimulation in real-time. 

## Figures and Tables

**Table 1:** Clinical characteristics of dystonia patients undergoing deep brain stimulation. SD: standard deviation.

Characteristics	Mean +/−SD	Range
Age	12.2 +/− 2.6	7–17
Sex (men/women ratio)	18/10	n/a
Weight (kg)	42.8 +/− 13.4	26.0–86.4
Length of anesthesia (min)	297.4 +/− 69.5	125–434
Length of surgery (min)	167.2 +/− 57.5	65–313

**Table 2:** Administered medications. The first two columns display the number (#) of cases and doses of medications (Dex: Dexmedetomidine) given during the entire procedure (MRIs + DBS), while the last two columns display these values for the intraoperative DBS part of the procedure (DBS only).

Medications	# cases	Dose	# cases	Dose
Dose	(MRIs + DBS)	(MRIs + DBS)	(DBS only)	(DBS only)
Propofol infusion given	11	n/a	7	n/a
Dex infusion given	14	n/a	14	n/a
Scalp block performed	7	n/a	7	n/a
Propofol (total, mg)	27	258.6 +/− 189.0 (40–673.5)	24	115.2 +/− 91.3 (26.7–271.7)
Dex (*μ*g)	14	36.7 +/− 30.4 (1.9–115.2)	14	33.9 +/− 23.8 (1.9–77.6)
Fentanyl (total, *μ*g)	18	113.6 +/− 102.9 (10–450)	15	37.7 +/− 24.4 (12.5–100)
Midazolam (mg)	15	2.1 +/− 1.8 (0.5–8.0)	6	0.6 +/− 0.2 (0.5–1.0)
Plasmalyte-A (mL)	24	954.2 +/− 581.4 (250–2500)	n/a	n/a

**Table 3:** Intraoperative complications. Intraoperative complications are shown. For all the 28 cases, the only intraoperative complication was a venous air embolism in one patient.

Intraoperative complication	All cases
Airway difficulty	0 (28)
Respiratory depression	0 (28)
Desaturation/hypoxemia	0 (28)
Bradycardia	0 (28)
Agitation	0 (28)
Conversion to gen. anesthesia	0 (28)
Seizures	0 (28)
Venous Air embolism	1 (28)

## References

[1] Fahn S, Bressman SB, Marsden CD (1998). Classification of dystonia. *Advances in Neurology*.

[2] Marsden CD, Obeso JA, Zarranz JJ, Lang AE (1985). The anatomical basis of symptomatic hemidystonia. *Brain*.

[3] Merello M, Cerquetti D, Cammarota A (2004). Neuronal globus pallidus activity in patients with generalised dystonia. *Movement Disorders*.

[4] Burke RE, Fahn S, Marsden CD (1986). Torsion dystonia: a double-blind, prospective trial of high-dosage trihexyphenidyl. *Neurology*.

[5] Lin J-J, Lin S-Z, Chang D-C, Ondo W, Jankovic J (1999). Pallidotomy and generalized dystonia. *Movement Disorders*.

[6] Lozano AM, Kumar R, Gross RE (1997). Globus pallidus internus pallidotomy for generalized dystonia. *Movement Disorders*.

[7] Obeso JA, Olanow CW, Rodriguez-Oroz MC, Krack P, Kumar R, Lang AE (2001). Deep-brain stimulation of the subthalamic nucleus or the pars interna of the globus pallidus in Parkinson’s disease. *New England Journal of Medicine*.

[8] Volkmann J, Sturm V, Weiss P (1998). Bilateral high-frequency stimulation of the internal globus pallidus in advanced Parkinson’s disease. *Annals of Neurology*.

[9] Lyons KE, Pahwa R (2004). Deep brain stimulation and essential tremor. *Journal of Clinical Neurophysiology*.

[10] Diamond A, Shahed J, Azher S, Dat-Vuong K, Jankovic J (2006). Globus pallidus deep brain stimulation in dystonia. *Movement Disorders*.

[11] Loher TJ, Hasdemir MG, Burgunder J-M, Krauss JK (2000). Long-term follow-up study of chronic globus pallidus internus stimulation for posttraumatic hemidystonia. *Journal of Neurosurgery*.

[12] Ostrem JL, Starr PA (2008). Treatment of dystonia with deep brain stimulation. *Neurotherapeutics*.

[13] Binder DK, Rau GM, Starr PA (2005). Risk factors for hemorrhage during microelectrode-guided deep brain stimulator implantation for movement disorders. *Neurosurgery*.

[14] Hsu Y-W, Cortinez LI, Robertson KM (2004). Dexmedetomidine pharmacodynamics—part I: crossover comparison of the respiratory effects of dexmedetomidine and remifentanil in healthy volunteers. *Anesthesiology*.

[15] Rozet I, Muangman S, Vavilala MS (2006). Clinical experience with dexmedetomidine for implantation of deep brain stimulators in Parkinson’s disease. *Anesthesia and Analgesia*.

[16] Ard J, Doyle W, Bekker A (2003). Awake craniotomy with dexmedetomidine in pediatric patients. *Journal of Neurosurgical Anesthesiology*.

[17] Maurtua MA, Cata JP, Martirena M (2009). Dexmedetomidine for deep brain stimulator placement in a child with primary generalized dystonia: case report and literature review. *Journal of Clinical Anesthesia*.

[18] Hooper AK, Okun MS, Foote KD (2009). Venous air embolism in deep brain stimulation. *Stereotactic and Functional Neurosurgery*.

[19] Herrick IA, Craen RA, Gelb AW (1997). Propofol sedation during awake craniotomy for seizures: patient-controlled administration versus neurolept analgesia. *Anesthesia and Analgesia*.

[20] Piccioni F, Fanzio M (2008). Management of anesthesia in awake craniotomy. *Minerva Anestesiologica*.

[21] Hans P, Bonhomme V, Born JD, Maertens De Noordhoudt A, Brichant JF, Dewandre PY (2000). Target-controlled infusion of propofol and remifentanil combined with bispectral index monitoring for awake craniotomy. *Anaesthesia*.

[22] Sinha P, Koshy T, Gayatri P, Smitha V, Abraham M, Rathod R (2007). Anesthesia for awake craniotomy: a retrospective study. *Neurology India*.

[23] Maze M, Scarfini C, Cavaliere F (2001). New agents for sedation in the intensive care unit. *Critical Care Clinics*.

[24] Ebert TJ, Hall JE, Barney JA, Uhrich TD, Colinco MD (2000). The effects of increasing plasma concentrations of dexmedetomidine in humans. *Anesthesiology*.

[25] Mack PF, Perrine K, Kobylarz E, Schwartz TH, Lien CA (2004). Dexmedetomidine and neurocognitive testing in awake craniotomy. *Journal of Neurosurgical Anesthesiology*.

[26] Pinosky ML, Fishman RL, Reeves ST (1996). The effect of bupivacaine skull block on the hemodynamic response to craniotomy. *Anesthesia and Analgesia*.

[27] Hartley EJ, Bissonnette B, St-Louis P, Rybczynski J, McLeod ME (1991). Scalp infiltration with bupivacaine in pediatric brain surgery. *Anesthesia and Analgesia*.

[28] Nguyen A, Girard F, Boudreault D (2001). Scalp nerve blocks decrease the severity of pain after craniotomy. *Anesthesia and Analgesia*.

[29] Ard JL, Bekker AY, Doyle WK (2005). Dexmedetomidine in awake craniotomy: a technical note. *Surgical Neurology*.

